# Rejoinder: multiculturalism and interculturalism: alongside but separate

**DOI:** 10.1186/s40878-018-0090-6

**Published:** 2018-06-19

**Authors:** Ricard Zapata-Barrero

**Affiliations:** Department of Political and Social Sciences, Universitat Pompeu Fabra, Barcelona, Catalonia Spain

**Keywords:** Interculturalism, Diversity, Post-multicultural, Proximity, Public space, Cities, Diversity-advantages

## Abstract

This rejoinder reacts to the comments I have received of my defence of interculturalism (key-article of this Special Issue). Basically it defends the need to take seriously the distinctiveness between MC and IC, as friends rather than foes. It is also argued that the emergence of IC must be placed in the context of legitimacy crisis of MC and the process of policy paradigm change and formation. Then, it is briefly stated that IC tries to fill the epistemological limits of MC and must be considered as a mainstreaming policy within the “local turn” in migration and diversity studies. Moreover, it is contended that IC is a new public mindset and announces a new public culture in a society of multiple-identities. Finally that IC makes diversity workwith a view of diversity as an advantage (which means that it is policy resource for cultivating community cohesion, creativity, economic development, solidarity promotion, xenophobia reduction). Finally, I reckon that IC probably requires a multidimensional theory of contact and a more deep normative reflection in terms of public benefits.

## Introduction: keeping distinctiveness between multiculturalism and interculturalism

Let me first thank all the contributors of this Special Issue for taking the time to articulate a reaction to my defence of interculturalism (IC) (Zapata-Barrero, 2017a). It seems to me that the fact that CMS has aroused such avidity is a symptom that something new is emerging out of this debate brewing interest within migration studies. For me this Special Issue is governed by two different guiding questions: a) whether IC constitutes a distinct policy paradigm; b) whether IC replaces multiculturalism (MC). After carefully reading all the contributions, including Modood’s key article, I feel that there has been some misunderstandings from the very beginning. In my defence, I thought my position was clear: *IC has the proprieties of being a new policy paradigm, but it does not replace MC*. Let me now emphasize that IC can contribute to renovate the initial MC project with more pragmatism, within which I have framed the post-multicultural period (post-M), and that MC could be a favourable factor in insuring the equality conditions for IC. I then agree with de Waal (2018); Levrau (2018) and others when they insist that both paradigms can be (need to be) implemented simultaneously. Of course, this would depend on the context and require a more accurate situational analysis, but the general trend can be drawn. In fact, I cannot imagine today one policy paradigm without the other. This statement obviously has epistemological consequences, as I will argue later. Multiculturalism can be right in certain circumstances, but not for whatever claim for recognition of difference. This awareness is what frames this post-multicultural period. The complementariness between MC and IC means that their mutual relationship can help attaint their different purposes and provide answers to their different needs in order to manage diverse societies. Each one can improve the qualities of the other. Maybe the concept of “interdependence” may draw a better picture of their relation. But everything depends on concrete situations and context which cannot be explored now in depth in this Rejoinder.

Taking into account these first premises, I would like to now address several questions raised by the different commentators. My arguments will be necessarily synthetic, given the length restraints of this rejoinder, but they will help map the basic points founding my position.

## Legitimacy crisis of MC, and the process of policy paradigm change and formation

Foremost, it is quite surprising that most scholars (even more so if we suppose they are diversity-sensitive) fall into the erroneous trap of focusing this debate on fusing “difference” with “opposition”. The fact I defend that both paradigms are distinct does not insinuate they are enemies, as if they were publicly offering a *querelle*, as Joppke (2018) seems to be happy to recreate. This *incommensurability hypothesis* is simply academical nonsense and can only be detrimental for migration studies. I also agree with those that warn us that this way of focusing the MC/IC debate can only fuel defenders of traditional assimilationism and radical neo-nationalists and xenophobe narratives.

I reckon that this *incommensurability hypothesis* has probably been one of the greatest mistakes coming from the “intercultural manifesto” White Paper of the Council of Europe (2008), and by some preliminary works coming from European policy-oriented “cookers” such as Wood and Landry (2008) and Cantle (2012), among others, but I have never fully endorsed this view. This initial interpretation needs to be incorporated within the same temporary process of continuities and change which I have drawn in my defence. It needs to be contextualised as the first necessary step of the process of IC policy paradigm formation. The context for me is also clear: the MC original policy paradigm meets two main interrelated challenges today, which has led it to a crisis of legitimacy. The first of the challenges is that after some two decades of application, and whatever gradual evidence-based interpretation we may draw, the overall diagnosis is that its rights-based approach has not reached, in most contexts, the equal and just society it pretended to attain. In some cities diversity is still seen related to a lack of trust and social capital, it is still considered a factor of inequality, power and unethical treatment. This *corroding effect* is far more evident in lower social class neighbourhoods, where social class conflicts become more visceral when identity variables penetrate public narratives, fueling racism and xenophobia. The second challenge is that MC struggles to provide answers to new diversity-related challenges, because in its original form, these factors where simply not in its agenda, as we will see in the next paragraph.

The premise of IC is evidence-based. There is a lesson to be drawn from this failure. Within this MC legitimacy crisis we are witnessing a process of policy paradigm change. This shift is coming from inside and from outside MC. From inside, MC now seems to be more aware of its limits, in front of the original “everything goes” in the process of recognition of difference, rights distribution and basic structural changes. From the outside, in my defence I have identified some new contextual factors in the migration and diversity research agenda: the securitisation framework that has penetrated the thinking of most diversity-management policies, preventing more open, cosmopolitan and humanistic policies. Other new social dynamics we can infer from diversity dynamics, such as super-diversity, transnationalism and multiple-identities, seem to be the norm in our mobile and diverse societies. The category popularized by Vertovec (2015) of super-diversity challenges most of the traditional ways we have debated the management of diversity. The racial view is just one way to interpret conflict in diversity contexts, but not the only one. This post-racial view of diversity, so as it has been called, incorporates intersectionality in diversity studies. It emphasizes the view that it is impossible to categorize the population in only ethnic and racial terms without taking into account other categories of diversity that may interact (gender, status, education, sexual orientation, and so on). MC faces difficulties in adapting to these new debates in the migration and diversity research agenda. Even the renovated neo-nationalist ideology which has become incorporated by mainstream politics fosters negative narratives on diversity that MC is unable to reduce.The outcome of this reviewing process is clear to me: the need for MC to do its own pragmatic turn, going from an ideal-MC to a much more realistic-MC period. In this post-MC context, IC acts as a pressure factor as it begins to sketch a separate legitimate policy paradigm that tries to address most of the deficiencies of the original MC project. This pragmatism needs to go from a group-based to an individual-based view of diversity management. This may perhaps come closer to the liberal tradition, in spite of the fact that making coherence between liberalism and MC were one of the first challenges that preoccupied the first MC “cookers” (Kymlicka, Taylor, Parekh, among others). In this process of policy paradigm change and formation, IC can give MC some sense of renovated continuity, but also, importantly, forcing MC to reboot itself, with the consequence of having to accept a change of status. MC must abandon its normative universalist ambition and accept it can only be a piece within a larger diversity management system, which is apparent that we need to re-construct. IC is forcing MC to get out of its finalist function and accept that it is a part of the social gear created by the dynamics of diversity. In this realistic process introduced by IC, MC becomes a means that may favour, in certain circumstances, the conditions of equality, meanwhile IC needs to promote positive contact, thus working together and complementing each other’s function.

We are in front of a process of policy paradigm change and formation, and it is normal that the defenders of the former paradigm (as Modood seems to be bunkerized) try to justify their master narrative and even subsume the new paradigm within their own, in some sort of natural academic biological reaction. I would even say that this has been the general trend of the MC narrative process. It incorporated one of the first critiques: cultural essentialism (Barry, 2000). There came other assessments, such as  the need to incorporate redistributive dimensions into the recognition dynamics (Fraser & Honneth, 2003), and so on. Now, this “bio-trend” must stop! It is true that IC comes to life only as a critique of multiculturalism (Modood, 2017; Joppke, 2018; Kastoryano, 2018) among others, but this “reaction” is a normal attitude announcing a process of policy paradigm changes and the formation of a new one (Hogan & Howlett, 2015). To focus a critique only on this specific stretch of a process is really surprising for scholars who usually work from a wide angle. In its turn, we should observe (or remember) that MC also emerged as a critique to assimilationism, but now coexist!

We are simply at the beginning of a process where the new paradigm (IC) is becoming institutionalized by policy makers, politicians, and it is further being academically accredited by a great variety of expert scholars. To cross out IC as a mere slogan (Joppke, 2018) is, in my view, an illustration of a lack of historical mindset. Additionally, the fact that Kastoryano (2018), for instance, devotes one section in her contribution following this rhetorical criticism seems a bit surprising to me, as she knows how important narratives are in understanding policy transformations, and how important the interrelations are between ideas, discourses and policies to form a policy paradigm. It is precisely this focus in which my efforts were concentrated in my defence. Some commentators have identified this process in my key-text, but I am unsure that all have done so.

## Epistemological limits of MC

I obviously endorse the basic MC *mantra*, its claims for equality and rights, for diversity-governance, diversity-incorporation, diversity-representation and diversity-participation, but I also expressed myself as a critic to what I have termed *boundless multiculturalism*. Through this expression, my purpose was to expose how naïf the original goodness tendency was in accepting whatever cultural differences derived from migrants without the strict filter of liberal and democratic values, of community cohesion and political stability.

MC still suffers from several pre-conceptions that IC could alleviate by critically drawing out underlying assumptions. In my defence I have highlighted the epistemological problems a national-based view of culture may cause, but also this tendency to have an uncritical group-based view of diversity, and on perpetuating the power-relation and inequalities that are substantial to diversity, keeping as theoretical framework the Unity-Majority-us and Diversity-minorities-them divide. The awareness of these “multicultural idols”, as I have labelled them elsewhere (see Zapata-Barrero, 2017b, pp. 169–193) is only possible when MC begin to recognise some premises coming from the IC emerging policy paradigm. This is probably one of the main analytical roles that IC is playing, contributing to braise the internal MC debate. In some sense, it is my conviction that keeping its distinctiveness as a policy paradigm, IC can impregnate MC of pragmatism. Without abandoning its ideals of equal justice, but with a clear feeling that rights-recognition have its limits. Let me now overview some of the substantial comments I have received, which will help me stress the proper place of IC as a distinctive policy paradigm in the current diversity debate.

## IC as a mainstreaming policy

My concern that MC has only been thought to target non-nationals is also not denied by the contributors, neither have they denied that the IC policy scope is broader as it also includes nationals. In fact, this mainstreaming nature of IC reinforces its intersectional and inter-public spaces dimensions. At this point multiculturalists need to be honest with themselves: MC was never thought as a policy to be relatable for citizens and nationals, but it was though out for those that have been interpreted as being “worst-off” in our society for legal, political, social and cultural reasons. The normative concern for justice in diverse societies impregnates this rawlsian interpretation of diversity in the original MC policy project. It is not surprising that the first multiculturalists were academically socialized with the debate of a just society of the 80s and the liberal/libertarian/communitarian debates that this generation aroused. We probably need a deeper exploration on how the theory of justice of the 80s influenced the initial MC project, maybe following the path of Levrau’s contribution. MC has been driven by an assumed principle of compensation of those that are seen as “different” from the mainstream. The view of ‘different’ and ‘worst-off’ have also been assumed, and has taken, in its radical form, a goodness attitude that has not contributed to appease the criticisms of its detractors. Within these preconceptions (here we are again!), MC has built its unity/diversity, majority/minority theoretical frameworks, separating, rather than bridging the ‘us’ and ‘them’ population, and even constructing this assumed power relation narrative of “privileged majority”. In my view IC solves some of these epistemological MC restraints. IC and mainstreaming policy presents many elective affinities as I have recently argued elsewhere (Zapata-Barrero, 2018a).

Behind this issue, there is the well-known diagnosis that in keeping separations between us/them, MC has legitimated specific policies for a ‘worst-off-them’ against a ‘well-off-us’. The question that IC answers is that it is possible to create diversity policies that follow equality and the recognition principles, without necessarily being group specific, but rather incorporating all people as a target public. I am sorry, but some contributors are still, including Modood, captured by this pre-conception, that we need to uncover to go a step further in this debate.

From an epistemological point of view, this pragmatic turn in the diversity debate announced by the emergence of the IC policy paradigm also highlights some key differences that some commentators have raised. The fact that diversity cannot be confronted with a national-based view of Unity. The function of the Unity category in social and political science has been twofold: the first is functional and ideologically-free: roughly speaking, Unity is what a society needs to share to insure stability and a certain degree of cohesion, a common understanding of the rule of law, shared democratic and liberal values and principle, human rights, and so on. But the fact that this has been mixed with national tradition and identity gives rise to a less ideological-free view of Unity. One outcome of this confusion is that Unity becomes viewed as “national majority”. In fact, this is one of the pillars of the xenophobic narrative and the national politics revival, and the basis where the duties-based approach of national civism has been constructed these last years (see my defence). Unity as a common public culture does not necessarily involve a power relation between a national-majority and a diverse-minority. One feature of our super-diverse societies is precisely that it is now very difficult to draw a diversity policy under the divisions of the population assumed by these frameworks. Everybody belongs to diversity and no one is the owner of a national Unity. Diversity is you and me, them and us. There is nothing beyond diversity. We need to go beyond this ‘magical’ tendency in which those that define diversity never include themselves within! IC is just inviting us to think of diverse societies from other vantage points, without abandoning its claims for equality and power sharing, and of course it is diversity-friendly, against some commentators casting doubt on this.

## IC and the “local turn” in migration and diversity studies

The nuclear idea of IC is that contact between different people is politically and socially as relevant as the institutional recognition of cultural differences. This move from macro-national to micro-local politics is at the foreground of what has been labelled the “local turn” we are witnessing in migration studies (Zapata-Barrero, Caponio, & Scholten, 2017). IC belong to this trend in the research migration agenda. Indeed, it is at the local level where the difficulties in implementing *boundless multiculturalism* arise. It is from this micro-level that most of the criticisms toward MC are drawn. In fact, we can adventure that in these concrete micro-circumstances MC has even fuelled the same conflict it tried to solve. As several commentators have righty identified, MC is implemented at the basic structure of society level, it is a state-based theory. This can explain why it has always focused on diversity-management using basic state tools, in terms of rights recognition and redistribution. In my defence, I have shown how recent works on multicultural indicators confirm this macro-view. It may seem that MC has always operated in terms of what Wimmer and Glick (2002) have categorized as “methodological nationalism” in which the nation-state is naturalized and understood as a container encompassing a culture, a polity, an economy and a homogeneous majoritarian group.

Within the section discussed above, I am sorry to have created some sort of confusion for Boucher and Maclure, as my position is not a “manifesto” against the State. It is of course necessary that the State deploys all its function to keep stability and cohesion within liberal democratic parameters. I am not criticising this role, but rather the epistemological consequences behind this state-based MC lens. This vantage point marks a great difference between the MC and the IC policy paradigm, as the latter has emerged ground-up from the cities. This “local intercultural turn” has some features on governance that have been identified by almost all the commentators: neighbourhood policies, face-to-face relations, public-sited spaces, proximity, and so on. I would say that even if it is not its unique sphere of application, public spaces play a central role for the IC policy lens, and it is in my view one of the forgotten areas of MC. In fact, for IC public space provides a critical setting for intercultural contact. Streets, squares, parks and markets provide the appropriate social conditions to reduce prejudices and build knowledge among people from different cultures, including national citizens. It also ensures the best conditions of solidarity promotion (Oosterlynck, Schuermans, & Loopmans, 2017). MC has difficulties in reaching these micro-views, because it has been constructed from university-laboratories rather than from the streets or with a local policy-maker mindset. It has further deployed all its policy engineering to accommodate differences within the basic structure, following mainly a rights-based approach. To put it in other words, it seems to me that the MC paradigm fails to see the role that municipal policies can play in setting up physical spaces that are conducive to intercultural contacts, which are likely to generate social cohesion, social capital and mutual understanding, paraphrasing Boucher and Maclure. In the following section, allow me to dive deeper with this line of argumentation.

## Interculturalism as a new public mindset, announcing a new public culture in a society of multiple-identities

As Levrau (2018) has also rightly identified, we need to articulate the content of an ethos, or what I have named an “intercultural mindset”. Indeed, this path of reflection is fully connected to my view that it is not “diversity of cultures” which we need to focus on, but on how to give content to a “culture of diversity”. This basically means that people need to learn to live within diverse settings, as this context is new for all (for newcomers, for those living here for long, new generations, citizens, etc.). What young people learn from diverse contexts is not always positive. There is much resentment and feelings of being treated unequally. There is even a learning process as to live with small-scale everyday racism, and even the worrying trend to trivialize racist situations, fear on public spaces governed by violence, cultural harassments, and self-restrictions to go to certain public spaces. At this micro-level there are many social relations that simply are unseen by a MC macro-scope, and that are important for confirming the feeling of belonging, cohesion, etc. MC has failed to articulate convincing answers for these frequent micro-conflicts, most of them driven by pre-judgments, stereotypes, and false rumours, invading the people’s public space, influencing their attitudes towards immigrants, threatening trust between people.

In few words: Diversity is a context we need to learn to live with. This is why I argue that the first premise to allow contact is diversity-acceptance. All the presented situations can only be managed if we help people accept diversity as a context of equality and not of power, injustice and inequality. To accept diversity means, of course, and mainly for citizens, to accept to be part of this category, even if “we” are from the mainstream national tradition. It is this socialization process that IC tries to foster, completely absent in MC. The IC conviction is that it is only through contact that people can learn self-respect and mutual-knowledge. Diversity-recognition at this micro-level cannot be imposed from above but from below, it must be the outcome of intercultural practice. Social psychologists like Berry (2013) insist that greater contact leads to more positive mutual regard. IC claims that diversity-recognition made by the basic structure of the society is not enough; it must be build for and by citizens. Only individual diversity-recognition can pave the path of motivation for contact. IC is a policy strategy to teach people to live in-Diversity through contact promotion.

Viewing IC as a structure from which to draw a new public culture allows us to map a new conceptual system within the diversity debate. Each concept playing different functions within the gear of diverse public culture: intercultural mindset, intercultural ethos, intercultural public space; in other words, intercultural citizenship. From this standpoint, I agree with de Waal (2018) when he says that MC predominately considers questions related to the ‘multicultural state’ in terms of power sharing and inclusion, while IC focuses more on civil society and the virtues, attitudes, dispositions and knowledge individuals need to possess to be “intercultural citizens”. Behind this argument there is the rousseaunian civic idea that one is not born a citizen, that citizenship is made through attitudes, behaviours and practices. IC is viewed as a socialization process, intercultural citizenship is a learning process within diversity through contact. The idea of IC as the creator of public culture is then an additional distinctive element. Let’s review a practical example coming from common language. “MC public space” describe a bounded territory of the city occupied by a variety of cultural groups, but not a space of contact, of encounters, contained in the notion of “Intercultural public space”.

This last argument allows me to enter in the well-known cultural group/territory nexus (again!). At this point, I agree with Kastoryano (2018) when she states that in social reality many concentrated neighbourhoods have become spaces of tensions among communities. IC is not so concerned with territorialisation as a fact, but it is rather more concerned that this could be the outcome of an unequal distribution of diversity and become a direct restriction for contact. Maybe we can point here that a theory of restrictions of contact is as necessary as a contact theory. The fact that there is no contact is not by itself an issue for IC, but only if it determines that this is due to some external restrictions influencing the autonomy of people, and it is further related to power relations and unequal status. This is why one basic concern of IC is to work towards the contextual conditions that make contact possible, especially in those urban areas where culture has been strongly territorialised and mixed with already existing social class and socio-economic conflicts. This territorialisation of cultural groups can be one driver of prejudices. It is here that there is a growing interest in knowing more about stereotypes, as these are factors that prevent encounters.

Maybe, in speaking about territorialisation and multiple identity we cannot avoid entering in the discussion related to the interculturalism/transnationalism nexus. I share Kastoryano’s focus of transnationalism as a process of deterritorialisation of culture and nationality. I have tried myself to articulate some key-arguments in other works (Zapata-Barrero, 2018b). Here I would like to enter in discussion with Kastoryano (2018), who seems to assume (she accepts they are distinct, by the way) that MC and IC have difficulties dealing with new transnational practices. In my view, the link between IC and transnationalism is on “overlapping affinities”. That is, if the rough notion of transnationalism is to live with at least two national identities, to have at least a bi-national mind, then the intrapersonal dialogue of transnational people about how to deal with their own complex identities is, in itself, an intercultural dialogue. The embeddedness in more than one national culture fosters the development of intercultural skills, namely, the capacity to enter in contact with other people with different backgrounds on equal terms. In other words, the growing importance of people with multiple national identities affiliations (the basis of transnationalism) is a favourable context for promoting contact between people from different backgrounds, including national citizens (the basis of interculturalism).

## Making diversity work: the place of diversity as a category of analysis and as a policy

Let me continue with another great astonishment. One of the differences (which does not mean opposition, as we have already stated) between MC and IC, which for me is essential is how each policy approach interpret the same category of diversity. To my knowledge the diversity-advantage argument was incorporated into the policy agenda by Wood and Landry (2008). It is substantial to understand how interculturalism legitimizes the importance of contact and to make diversity work.

Contact here becomes the driver for promoting diversity-advantages, which basically means the use of diversity as a policy resource for cultivating some public benefits: creativity, social and economic development, community cohesion, community identity, sense of city belonging. The premise is clear: only within diversity can we create the conditions for living within diversity. This view of diversity as a resource is probably instrumental, because it originally comes from the initial approach on diversity of business, urban and even social psychology studies. All these approaches deal with diversity in terms of what it can produce in relation to benefits. As a policy paradigm, IC views diversity production as public benefits. The assumption is that contact can only produce positive outcomes if it is done under equal conditions and in the context of power sharing. This supposition becomes the premise for policy engineering. Evidence tells us that creative cities, economically developed cities in our global world are all-diverse and have taken diversity as a resource to produce positive outcomes. IC is a policy tool for this. It follows that IC does not have a direct interest in justice (as MC does), its interest lie on making diversity work. Without this view of diversity as advantage, IC would simply lose its normative meaning. It is much more than the simplification made by Joppke (2018), when he states that diversity is “business-minded” in interculturalism. The link between diversity, creativity and development is something that is absent in the MC normative project. In this sense IC becomes some sort of policy engineering since is seeks to promote contact as a way to cultivate a resource that could only have in short and long terms, public benefits at the individual and social levels. It is at this point where its normative force and its political attractiveness resides. To figure out what are the exact contents of the advantages when we “make diversity work” through contact promotion is probably one of the research areas that will require deep exploration in the coming years.

## We need an intercultural theory of contact

Let me finish this rejoinder with a complete agreement with Oosterlynck (2018). We need an intercultural theory of contact, since we cannot assume a one-dimensional view of contact or that all contacts can have the same effect. Of course, we could begin with Allport’s contact hypothesis, but this can only be theorized empirically. Contacts can have different intensities and grades, can be practised in formal and informal settings, in many different public spaces, with many purposes and expectations.

What Oosterlynck (2018) suggests that I should strengthen is precisely the need to widen the original notion of contact, and I would certainly place interdependency within this scope. Oosterlynck speaks about interdependency and struggle as source of solidarity, and we can even say that both are also drivers of interculturalism. More than struggle, I would say mobilisations for common interests. Of course, solidarity may arise in demonstration for legal rights, political rights, and for housing claims. It is here, where I would say that IC presents its most demanding face, since through mobilisations people interact and develop a sense of solidarity.

In this effort to theorize contact, there is some confusion that some commentators provoke, but also denounce. Boucher and Maclure (2018), for instance, have put the difference between contact and dialogue rightly, and I have maybe nothing more to say than to endorse their views and add some more dimensions within this analytical difference. The fact that they situate me in the interactionist view is maybe too thin to my broad perspective, however I reckon I have endorsed this in my previous writings. This is why I have chosen for my defence a “less nuanced” concept of contact, which can include interaction (when there are people following joined-actions), but also other degrees of contact, including interdependence and simple informal encounters such as in the market or in public gardens, but also while waiting for children in the school and at the doctors lobby. ‘Contact’ is action-driven and a context-based concept. ‘Dialogue’ implies some degree of negotiation between the two parts, which is not the case for contact. ‘Intercultural dialogue’ comes from international relations, and, in its origins it implies a strategy to solve ethnic conflicts at the global sphere. ‘Dialogue’ is a strategy for negotiation. The fact that Modood (2017) emphasizes that intercultural dialogue has been one issue within the MC agenda seems to contribute to this confusion. I am not sure he is aware of this. Intercultural dialogue is some sort of cultural *modus-vivendi*, which is absent within the more ‘neutral’ concept of contact.

Seeing IC as if it were a cultural identity negotiation processes, also seems to be the position assumed by Kastoryano (2018). This is a view of IC-as-dialogue we must reject. Indeed, IC promotes contact, but not with the purpose of offering some forum of negotiations amongst people in relation to their cultural identities. The view of contact-as-negotiation could only be seen in what I called the political and contractual strand of IC, the one that focuses on the link between Diversity, national and cultural traditions. But this negotiation is not done among people. It is the basic view of the Quebecois understanding of IC illustrated by Bouchard (2015). It is true that this vertical level still needs to be incorporated into the European debate, since most of the public discussions on Britishnes, Frechness are based on this process of negotiation between diversity and an essentialist view of cultural tradition and national identity, which is for me a much closer illustration of remaining romanticism. Who is British today or French “de souche” is a categorisation that needs to be relativized in super-diverse societies, reflecting more on virtual reality than on politically reproduced xenophobic narratives. As I have argued above, in my view MC fuels the latter narrative more than IC.

## To conclude: taking seriously the distinctiveness between MC and IC. Friends rather than foes

I would like to conclude calming the spirit of multiculturalists. They have no reason to be offended. I have argued that MC has many reasons to move from an initial idealist period to a much more pragmatic one, limiting its claim for inclusion under the auspices of equal justice. This MC pragmatic period (post-MC period) also means that the MC policy paradigm must abandon its hegemonic attitude and learn to share diversity-friendly narratives with other emerging distinctive policy paradigms: IC. In fact, I think that in this MC legitimacy crisis I have drawn, IC can play the role of giving MC new breath.

I strongly recommend abandoning this *incommensurability thesis* polluting the MC/IC debate, and to focus the discussion more constructively. I am convinced that working-together they have better conceptual and normative resources to reach their differentiated objectives: a just and equal diverse society but also a much more cohesive, stable and creative society. Once their distinctiveness is accepted, the next step must certainly be to analyse their interdependencies. Now I am speaking directly to Modood: to negate their distinctiveness could be a fatal error for the development of the debate, since keeping both paradigms analytically distinct can not only enrich the diversity and equality nexus debate, but it can also improve its resources and maximize its normative pretensions. Thus, to include IC as a MC trend could even be thought of as a symptom in which one has not yet overcome the fact that MC is not alone, that there are other legitimate political paradigms that can complete what MC cannot achieve. In my opinion, pretending to absorb IC within MC, as Modood (2017) suggests, is also a symptom that the much-needed pragmatic turn of MC still faces many difficulties driving a policy paradigm change. Multicultarists trivializing on IC is for me an illustration that certain “multicultural idols” still remain in this policy paradigm change period in which I have placed the debate.
